# Modeling Semantic Emotion Space Using a 3D Hypercube-Projection: An Innovative Analytical Approach for the Psychology of Emotions

**DOI:** 10.3389/fpsyg.2016.00522

**Published:** 2016-04-19

**Authors:** Radek Trnka, Alek Lačev, Karel Balcar, Martin Kuška, Peter Tavel

**Affiliations:** ^1^Science and Research Department, Prague College of Psychosocial Studies (PVSPS)Prague, Czech Republic; ^2^Faculty of Humanities, Charles University in PraguePrague, Czech Republic; ^3^Olomouc University Social Health Institute (OUSHI), Palacky University in OlomoucOlomouc, Czech Republic; ^4^Health Psychology Unit – Institute of Public Health, Medical Faculty, P. J. Safarik UniversityKosice, Slovakia

**Keywords:** emotions, emotional experience, affect, semantic, dimensions, circumplex model

## Abstract

The widely accepted two-dimensional circumplex model of emotions posits that most instances of human emotional experience can be understood within the two general dimensions of valence and activation. Currently, this model is facing some criticism, because complex emotions in particular are hard to define within only these two general dimensions. The present theory-driven study introduces an innovative analytical approach working in a way other than the conventional, two-dimensional paradigm. The main goal was to map and project semantic emotion space in terms of mutual positions of various emotion prototypical categories. Participants (*N* = 187; 54.5% females) judged 16 discrete emotions in terms of valence, intensity, controllability and utility. The results revealed that these four dimensional input measures were uncorrelated. This implies that valence, intensity, controllability and utility represented clearly different qualities of discrete emotions in the judgments of the participants. Based on this data, we constructed a 3D hypercube-projection and compared it with various two-dimensional projections. This contrasting enabled us to detect several sources of bias when working with the traditional, two-dimensional analytical approach. Contrasting two-dimensional and three-dimensional projections revealed that the 2D models provided biased insights about how emotions are conceptually related to one another along multiple dimensions. The results of the present study point out the reductionist nature of the two-dimensional paradigm in the psychological theory of emotions and challenge the widely accepted circumplex model.

## Introduction

Language is a primary tool of emotion research and the primary access to the affective experience of the self and others (Storm and Storm, 2005). The key importance of research instruments inspired in psycholinguistics is apparent when exploring the research designs of empirical studies in this field. Aside from some experimental (e.g., Gerdes et al., 2013; Grol and De Raedt, 2014; Yu et al., 2015) and observational research (e.g., Jensen, 2014; Rohlf and Krahé, 2015), a huge number of current empirical studies used linguistic properties when exploring the terrain of human emotional experience (e.g., Crutch et al., 2013; Bayer and Schacht, 2014; Gallant and Yang, 2014; Schindler et al., 2014). People construct and understand their emotional experience through the abstract representations of emotions in language. For this reason, it is not surprising that psycholinguistics attract many researchers who seek out the links between language, cognitive processes and emotional experience (Aznar and Tenenbaum, 2013; Fisher et al., 2014; Verhees et al., 2015). The present study follows just this line of research and investigates interrelations between the experiential component of emotions and the anchoring of emotional concepts in language.

First of all, it is necessary to say that human emotional life is fascinating due to its very high degree of complexity (Grühn et al., 2013). The terrain of human emotional experience seems to be a little bit complicated and non-transparent, which, on the other hand, attracts psychologists who are motivated to explore fields that are not simply structured. In contrast, we can sometimes see a tendency to shape research designs in a simplified manner in an effort to provide readers with clear solutions. The effort to present scientific results in a simple and structured form may even lead to a superfluous reduction of the phenomena. As an example, it is very difficult to approach the complexity of human emotions within only two general dimensions (Roberts and Wedell, 1994; Fontaine et al., 2007). The newest research findings on the global meaning structure of the emotion domain pointed out that more than two dimensions are needed to describe the nature of the human emotional experience sufficiently (Fontaine et al., 2007; Fontaine and Scherer, 2013). The present study was inspired by this current progress in emotion research and continues in the further development of a multidimensional approach to the study of human emotions.

At the beginning we will focus our attention on the conceptual embedding of psychological research of emotions. The general organization of the semantic field, linguistic labels and the conceptual structure actually attract the attention of researchers (Boutonnet et al., 2014; Kuehnast et al., 2014; Troche et al., 2014). A proper defining of various theoretical concepts is necessary at the beginning of our work.

Emotional meaning systems are culturally-specific systems that shape the ways in which people experience, express, organize, and modulate their emotions (Parkinson et al., 2005). Emotional meanings specifically are abstract devices that shape general emotion knowledge; they may be subjectively chosen by the cognitive processing of an individual from a collective emotional meaning system shared in a given culture.

Emotion concepts are abstract representations of experiences of various emotions in one's mind (Oosterwijk et al., 2009). They are components of the general emotion knowledge of an individual (Oosterwijk et al., 2009) that is formed by storing previously experienced sensory, motor, physiological and introspective states. Faucher and Tappolet (2008) distinguished three forms of knowledge about emotions: conceptual knowledge about emotions, personal knowledge about emotions and knowledge about others' emotions.

Some emotion concepts represent a group of feelings that is qualitatively different from other emotional experiences. They are called emotion categories (Russell and Lemay, 2000), emotion prototype categories (Reilly and Seibert, 2003) or prototypes (Parkinson et al., 2005). Specific emotion terms, emotion words or emotion names mean linguistic labels for emotion categories in language (Hupka et al., 1999). Mutual relations between the semantic fields of all emotion prototypes in one's mind define the overall structure of the semantic space for emotions (Scherer, 2005), also called the subjective emotion space (Sokolov and Boucsein, 2000; Trnka, 2013). In a similar vein, Reisenzein and Schimmack (1999) used the term “affect structure” as the constitutional makeup and interrelations between emotion prototypes. The subjective emotion space is not equally limited in each person, and its size is given by the maximum extremes and minimal minimums of the range of each of the possible dimensions of experience (Trnka, 2013). The extent of subjective emotion space may change throughout the life course, due to the process of evolution of emotion concepts and emotion prototypes over time (Scherer, 2005).

The fascinating question is what qualities does subjective emotion space have? The discussion about the dimensionality of human emotional experience is dynamic and long-lasting (see Scherer, 2013, for an overview). Currently, most empirical studies have utilized the two-dimensional circumplex model (Russell and Lemay, 2000) that includes only two general dimensions of valence and activation (Kuppens et al., 2013). Even as the most frequently used, the two-dimensional model faces several problems, especially, when one thinks about the positions of various discrete emotions within the dimensions of valence and activation (Roberts and Wedell, 1994; Trnka, 2013). To understand the complex structure and varieties of human emotional experience on two quite general dimensions is reductionist. The question is if it is possible to investigate complex emotions like shame, guilt, envy, or compassion within a simple two-dimensional paradigm working with dimensions of valence and activation? We offer to disagree. It can be said that the above-mentioned emotions are somehow pleasant or unpleasant and that they are somehow intense, but, in doing so many slight variations of such complex feelings remain hidden. Also, assessing the semantic similarity between complex emotions in the semantic space of individuals is problematic when using only the above-mentioned basic dimensions of valence and activation. We argue that using a simple two-dimensional model for an in-depth analysis of the human emotional experience may lead to a risk of reduction of the complexity of the phenomena.

Further, the critique of utilizing only a limited number of basic dimensions was not focused only on complex emotions, but on basic emotions, as well. Roberts and Wedell (1994) pointed out the high degree of reductionism when utilizing the dimension of valence and activation within the framework of simple multi-dimensional scaling (MDS) techniques. For instance, anger and fear are usually placed very near to each other in two-dimensional space, since they mean a high amount of arousal with a strongly negative valence (Russell, 1980; Watson and Tellegen, 1985; Larsen and Diener, 1992). This might lead to the impression that these two emotions are very similar to each other (Roberts and Wedell, 1994), yet in reality they both have their specific experiential character.

Given the above-mentioned arguments, it seems indispensable to think about further conceptual and methodological development in this field. One way of overcoming the limitations of the two-dimensional model for assessment of the complex structure of emotional experience is to explore some possibilities of a multidimensional conceptual embedding of emotion research, for example, as proposed by the theory of multi-dimensional emotional experience (Trnka, 2013).

Sokolov and Boucsein (2000) pointed out that the general preference of researchers for a reduction of emotion space to a small number of dimensions is probably influenced by attempts to visualize such a dimensional system within familiar Euclidian geometrical space. However, the current inductive study of Fontaine and Scherer (2013) showed that the global meaning structure of emotions can be optimally described by four dimensions: valence, arousal, power/control, and novelty. In a similar vein, Sokolov and Boucsein (2000) introduced an alternative, four-dimensional theoretical model for approaching subjective emotion space. They showed that discrete emotions can be analyzed on a hypersphere in four-dimensional space. The concept of a hypersphere probably provides a less-reductionist paradigm for the investigation of human emotional experience.

Actually, it is not clear how many and what kinds of dimensions could be optimal for the construction of a hyperspace that would fit well for the analysis of human emotional experience. Empirical studies employing more than two-dimensional solutions of emotional experience are almost lacking, with some exceptions (Fontaine et al., 2007; Fontaine and Scherer, 2013). Therefore, the field is now open for new, experimental work exploring various multidimensional approaches in the psychological research of emotions. This was also the challenge and the starting point for the present study.

## Aims and significance

The present study brings further knowledge to a new, developing field of multidimensional research in the psycholinguistics of emotions. We challenge the use of the widely accepted theoretical two-dimensional circumplex model working only with the dimensions of valence and activation. The goal of the present study was not to solve the question of how many dimensional qualities subjective emotion space actually has but to examine the use of a new model working with more than two dimensions in the analysis of the overall structure of the semantic space for emotions. More specifically, we used four different dimensional measures as input data for this model. We hypothesized that all four input dimensional measures will be uncorrelated and, therefore, can be considered as clearly different qualities of subjective understanding of emotion prototypes. We follow current progress in psycholinguistics (Fontaine and Scherer, 2013) and introduce an innovative analytical approach for emotion research working in other than the conventional, two-dimensional paradigm.

We focused the study on methodological improvements in data gathering that fit well for the assessment of slight differences in the mutual positions between various emotion prototypes in the participants' judgments (see the Materials and procedure subsection for more details). The main goal was to construct a three-dimensional model based on this data. The main output of the present study is, therefore, the construction of a 3D hypercube-projection including the positions of the main emotion prototypes as the basic constitutive elements of semantic emotion space. Various aspects of the constructed 3D hypercube-projection and limitations of the proposed methodological approach are discussed in the final part of the study.

## Methods

### Subjects

Participants were 187 university students with an age ranging from 19 to 38 years (*M* = 22.6; *SD* = 3.2). Sex distribution was 54.5% female. All participants were Czech native speakers and participated voluntarily in the study. The research design was approved by the institutional ethics committee and also by the principal governmental research institution, the Czech Science Foundation. All participants signed the informed written consent with their participation in the study.

### Materials and procedure

At the beginning, participants filled in the basic demographical characteristics. Following a discrete emotions paradigm (Kunzmann et al., 2014), 16 emotion words were judged by participants: anger, fear, sadness, happiness, disgust, hope, love, hate, contempt, guilt, compassion, shame, gratefulness, envy, disappointment, jealousy. These emotion prototypes cover five basic emotions (anger, fear, sadness, happiness, disgust) as well as complex emotions like hope, love, hate, contempt, guilt, compassion, shame, gratefulness, envy, disappointment and jealousy. The whole judging procedure was conducted in the Czech language. All of the emotion words used were non-synonymous.

Participants judged all 16 emotion words four-times. A separate list including the 16 above-mentioned emotion words was provided for each judgment. Each of four judgments measured subjective understanding of the provided emotion words on another dimension. First, participants judged all 16 emotion words on the dimension of valence. A 10 cm horizontal line was provided next to each of the 16 emotion words. Participants were asked to rate the degree to which they experienced this emotion as pleasant/unpleasant using the instruction: “Please, mark on the following lines how much you experience the particular emotion as pleasant or unpleasant.” The participants then marked the position of each emotion word on the 10 cm lines provided next to each of the 16 emotion words. The same tool was also used in the following three judgments. Second, the participants judged the same 16 emotions on the dimension of intensity introduced by the instruction: “Please, mark on each line how much you experience a particular emotion as calm or aroused.” Third, the same 16 emotion words were rated on the dimension of control. Participants were provided with the instruction: “Please, mark on each line how much you are able to control a particular emotion in the sense that it does not influence your thinking or behavior”. Fourth, the same 16 emotion words were rated on the dimension of utility using the instruction: “Please, mark on each line how much you perceive that the following emotions are harmful or beneficial for you?” A separate sheet was used for each of the four judgments.

The used measurement was dimensional, but the data obtained from the participants' judgments will be called “aspects” or “input dimensional aspects” throughout the remaining part of the paper. This change was made to provide a clear differentiation between dimensional data entering the analysis and the dimensions that were constructed in the course of data analysis.

### Data analysis

The above-described methodological instrument for data gathering enabled a fine-grained assessment of slight differences in the mutual positions between the semantic fields of various emotion prototypes in the semantic spaces of participants. By measuring the position of the marked points on the line segment, the millimeter positions from the left end were obtained, thus providing a scale from 0 to 100 (mm). These positions varying between 0 and 100 were then entered into the data analysis and were used for the construction of a 3D hypercube-projection. This assessment of semantic fields on a line segment is more fine-grained in comparison with standard Likert-type scales using five of seven non-continuous options. Therefore, the 3D hypercube-projection constructed based on this methodology captured the mutual positions of participants' judgments more accurately than projections based on data from a Likert-type scale.

To obtain the multidimensional emotional space and assess the necessary number of dimensions needed to properly explain the relations between the individual emotion prototypes, multidimensional scaling based on correlations between the individual emotions in each of the aspects was used. Multidimensional scaling is a useful tool to help understand people's judgments considering the similarity of members or objects and thus to produce inductive, but empirically derived “maps of elements.” In multidimensional scaling we try to find a configuration of points in space in which the distance between these points match as close as possible the original proximities between the objects (Busing, 1998). Thus, the MDS technique enables us to construct a 3D hypercube-projection based on the perceptions of a diverse set of individuals who are blind to the exact purpose of the give study. PROXSCAL with multiple matrices as the source (as provided by the statistical software SPSS 20) was used to examine dimensions within the data. This algorithm minimizes raw normalized stress, and thus the result is a far more “honest” Euclidean space. Compared with other existing scaling options PROXSCAL has a number of important advantages (Busing et al., 1997). As a source matrices for MDS Spearman correlation matrices—one for each aspect was created and transformed to proximities so that a high positive correlation meant high proximity, while a high negative correlation meant low proximity (i.e., *r* = 1 was transformed to distance 0, *r* = 0 was transformed to distance 1 and *r* = − 1 was transformed to a distance of 2, etc.).

## Results

### Descriptive data

The four originally measured aspects—i.e., valence, intensity, control and utility—were not significantly correlated (α = 0.05) when using Bonferroni correction for multiplicity. Table 1 includes the arithmetic mean (M) scores for each emotion in each aspect as well as its standard deviation (SD). Each emotion was transformed to values from 0 to 100 according to a mark in the answer sheet—where each 1 point corresponds with 1 mm distance from the left-side beginning of the line. Thus, for valence, the more the numbers drop below 50, the more unpleasantness they express and vice versa for numbers where a number reaching 100 means maximum pleasantness. The numbers for arousal (ranging from 0—calm to 100—aroused), control (ranging from 0—uncontrolled to 100—controlled) and finally utility (ranging from 0—harmful to 100—beneficial) function in a similar way.

**Table 1:** **Descriptive results of assessment of emotions (whole sample, *n* = 187)**.

**Emotion**	**Valence**		**Arousal**		**Control**		**Utility**	
	***M***	***SD***	***M***	***SD***	***M***	***SD***	***M***	***SD***
Anger	25.3	19.4	79.4	16.9	47.7	27.4	32.4	23.1
Fear	18.1	17.8	75.6	18.7	50.0	24.9	39.9	24.4
Sadness	17.4	17.3	47.1	26.6	49.9	25.6	36.1	21.9
Happiness	91.8	12.0	69.1	30.4	73.9	62.2	89.5	11.0
Disgust	26.1	17.5	53.8	21.9	60.4	22.6	35.9	16.8
Hope	77.1	17.4	53.6	28.1	71.7	20.4	79.5	16.9
Love	91.2	14.9	67.9	33.7	55.2	31.9	90.2	14.3
Hate	18.7	18.2	65.9	21.7	48.8	24.3	24.7	20.7
Contempt	22.1	17.8	51.6	43.8	57.2	23.7	27.4	17.8
Guilt	15.6	13.9	55.4	24.4	45.2	22.9	39.0	23.5
Compassion	51.4	20.6	39.1	21.0	63.4	20.8	64.5	19.7
Shame	24.3	15.9	60.6	20.9	45.5	23.7	40.9	20.8
Gratefulness	68.3	18.3	40.4	23.9	69.2	20.6	73.0	16.2
Envy	22.9	16.2	56.5	49.3	65.8	24.6	22.9	18.5
Disappointment	17.3	14.1	53.5	25.6	45.3	24.7	41.0	20.3
Jealousy	23.9	17.7	71.7	19.7	48.0	26.6	26.5	21.7

### Multidimensional scaling

To simply analyze proximities between emotions in these original four aspects we can construct a matrix using arithmetic means as coordinates in 4D space (using w-, x-, y-, and z-axis). To analyze proximities we assume the same weight of all aspects and thus use the simple distance (s) obtained by the following formula (where w, x, y, and z are the differences between the values of arithmetic means of two emotions in each of the four dimensions):

$$s=\sqrt{w^{2}+x^{2}+y^{2}+z^{2}}$$

The lower the number in the resulting proximity matrix, the closer the two emotions are in theoretical 4D space. The range of possible distances is (0–200) where 200 is the length of the hypotenuse of a theoretical hypercube with each side of length of 100. The distances are listed in Table 2.

**Table 2:** **Original emotional proximities in theoretical 4D space based on originally measured aspects**.

**Distance (s)**	**Fear**	**Sadness**	**Happiness**	**Disgust**	**Hope**	**Love**	**Hate**	**Contempt**	**Guilt**	**Compassion**	**Shame**	**Gratefulness**	**Envy**	**Disappointment**	**Jealousy**
Anger	11.3	33.6	92.1	28.8	78.4	88.7	16.9	30.0	26.9	59.9	20.8	74.1	30.8	28.6	9.8
Fear		28.8	92.3	25.8	77.5	89.3	18.0	28.3	21.0	56.9	16.9	72.4	25.3	22.7	15.2
Sadness			97.2	15.2	77.2	93.9	22.1	13.1	10.1	47.0	16.5	66.1	21.5	9.2	27.2
Happiness				87.2	23.8	18.8	100.9	96.5	96.8	57.2	88.4	40.9	114.0	94.8	96.2
Disgust					67.9	86.0	21.5	10.3	18.8	40.9	17.2	58.4	36.6	18.2	23.8
Hope						28.2	84.1	77.1	78.2	34.1	70.8	17.2	96.4	75.9	80.7
Love							97.9	94.8	92.6	56.0	84.0	42.0	114.2	90.5	93.0
Hate								17.2	18.4	59.9	18.2	76.6	31.7	20.8	8.0
Contempt									18.3	49.3	20.1	67.0	29.1	18.8	22.2
Guilt										50.2	10.3	68.8	44.7	3.2	22.3
Compassion											45.6	19.9	72.6	47.4	59.1
Shame												62.8	45.8	10.0	18.3
Gratefulness													87.6	66.1	74.6
Envy														28.0	23.6
Disappointment															24.3

**Figure 1:**
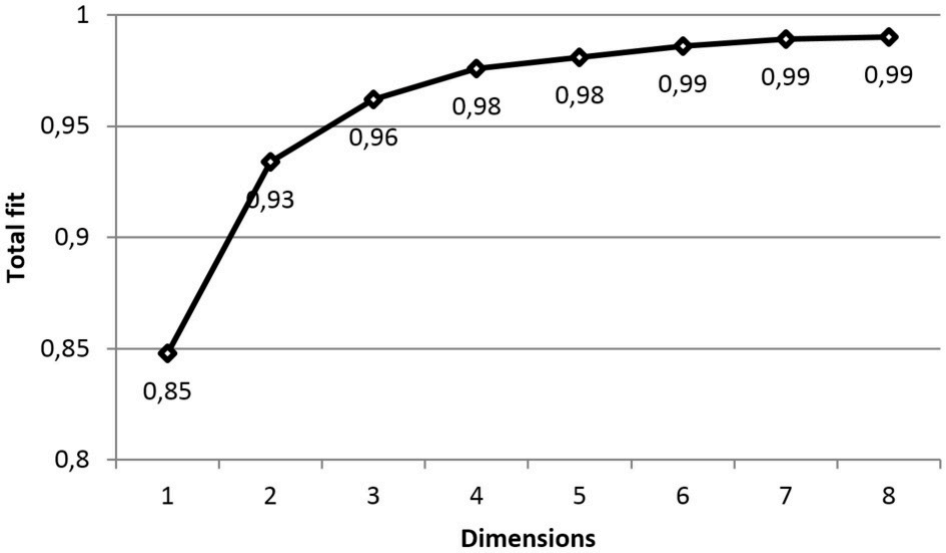
**Scree plot**. Total fit calculated according to Busing et al. (1997).

Following the descriptive analysis, we used the individual correlation matrices (as described above) for each of the original aspects and obtained PROXSCAL solutions for one through twelve dimensions. Based on these results (see Figure 1), a three-dimensional solution with a total fit of 0.96 was chosen, because the increase in the total fit by adding a fourth dimension was very small.

The values of stress obtained with a simplex start and the amount of variance accounted for by a three-dimensional solution are shown in Table 3. The iterations were stopped at 34 because the S-stress improvement was less than 0.001. However, there is no rule of thumb to interpret the quality based on the normalized raw stress results. Busing et al. (1997), Busing (1998) calculates the total fit by subtracting the total stress from 1. We can than borrow a rule of thumb from Kruskal (1964): 0.2 = poor; 0.1 = fair; 0.05 = good; 0.025 = excellent and 0.0 = perfect.

**Table 3:** **S-stress improvement (for a three-dimensional solution)**.

**Iteration**	**S-Stress**	**Improvement**
0	0.42697	
1	0.09666	0.33030
2	0.07608	0.02059
3	0.06733	0.00875
4	0.06154	0.00579
5	0.05736	0.00418
6	0.05421	0.00315
7	0.05170	0.00251
8	0.04964	0.00206
9	0.04792	0.00172
10	0.04648	0.00144
11	0.04528	0.00121
12	0.04427	0.00101
13	0.04342	0.00084
14	0.04272	0.00071
15	0.04212	0.00060
16	0.04161	0.00051
17	0.04118	0.00044
18	0.04080	0.00038
19	0.04047	0.00033
20	0.04017	0.00030
21	0.03991	0.00026
22	0.03967	0.00024
23	0.03946	0.00021
24	0.03926	0.00020
25	0.03908	0.00018
26	0.03891	0.00017
27	0.03876	0.00015
28	0.03862	0.00014
29	0.03848	0.00013
30	0.03836	0.00013
31	0.03824	0.00012
32	0.03813	0.00011
33	0.03802	0.00011
34	0.03792	0.00010

**Figure 2:**
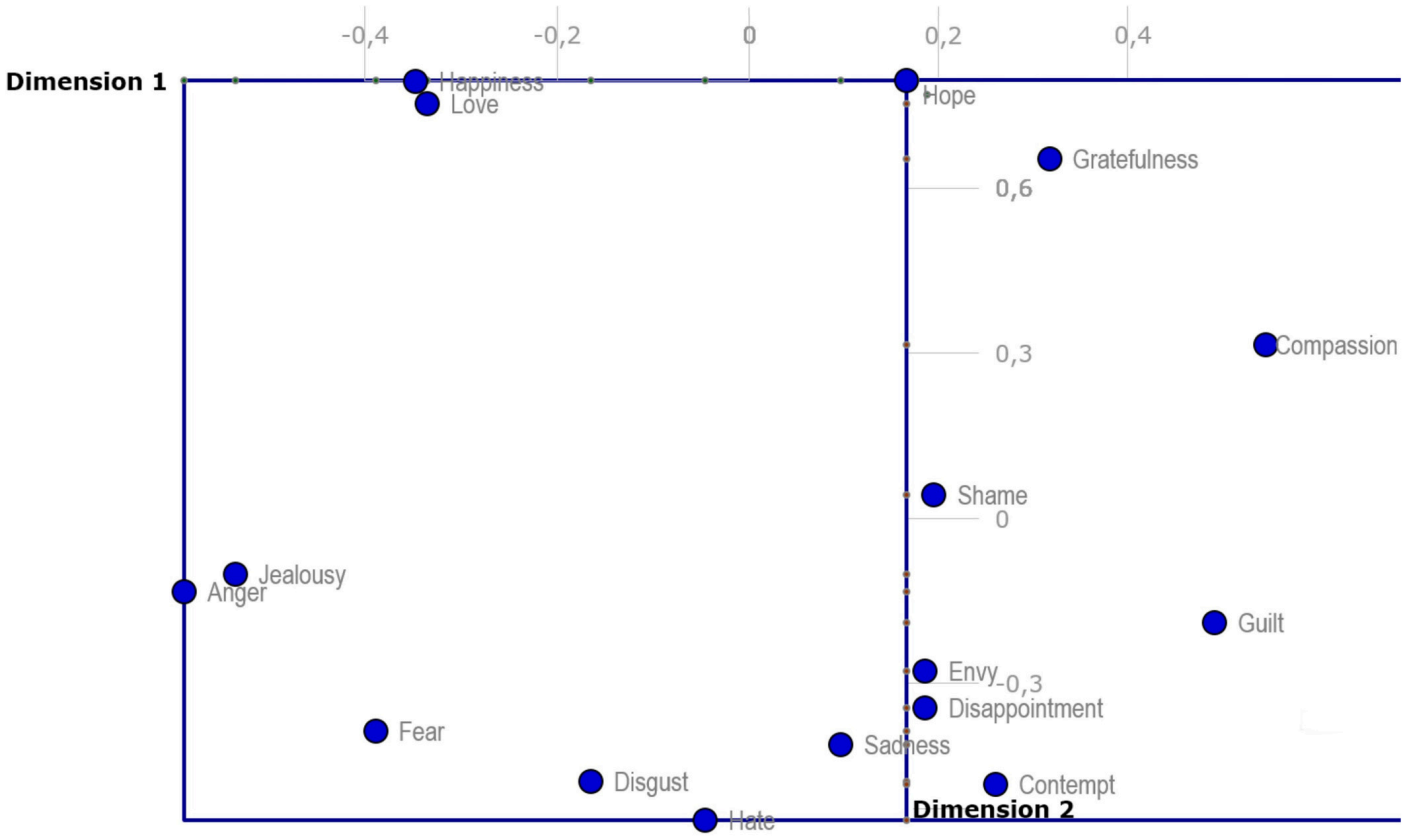
**Two-dimensional projection of discrete emotions on Dimension 1 and Dimension 2**.

A two dimensional (2D) representation of each of three resulting dimensions shows clear groups of emotions on three different planes. Figure 2 of the Dimensions 1 and 2 plane indicates that there are several clusters of emotions close together—happiness and love accompanied also by slightly more distant hope and gratefulness on one side of the plane, and anger and jealousy accompanied with slightly more distant fear on the other side of the plane; the third vertex of the imaginary triangle consists of a larger cluster of envy, disappointment, sadness and contempt accompanied also by the more distant hate, disgust and possibly also shame and guilt. Other emotions are somewhere in between these clusters (e.g., fear) or somewhat outside creating its own category, such as compassion.

**Figure 3:**
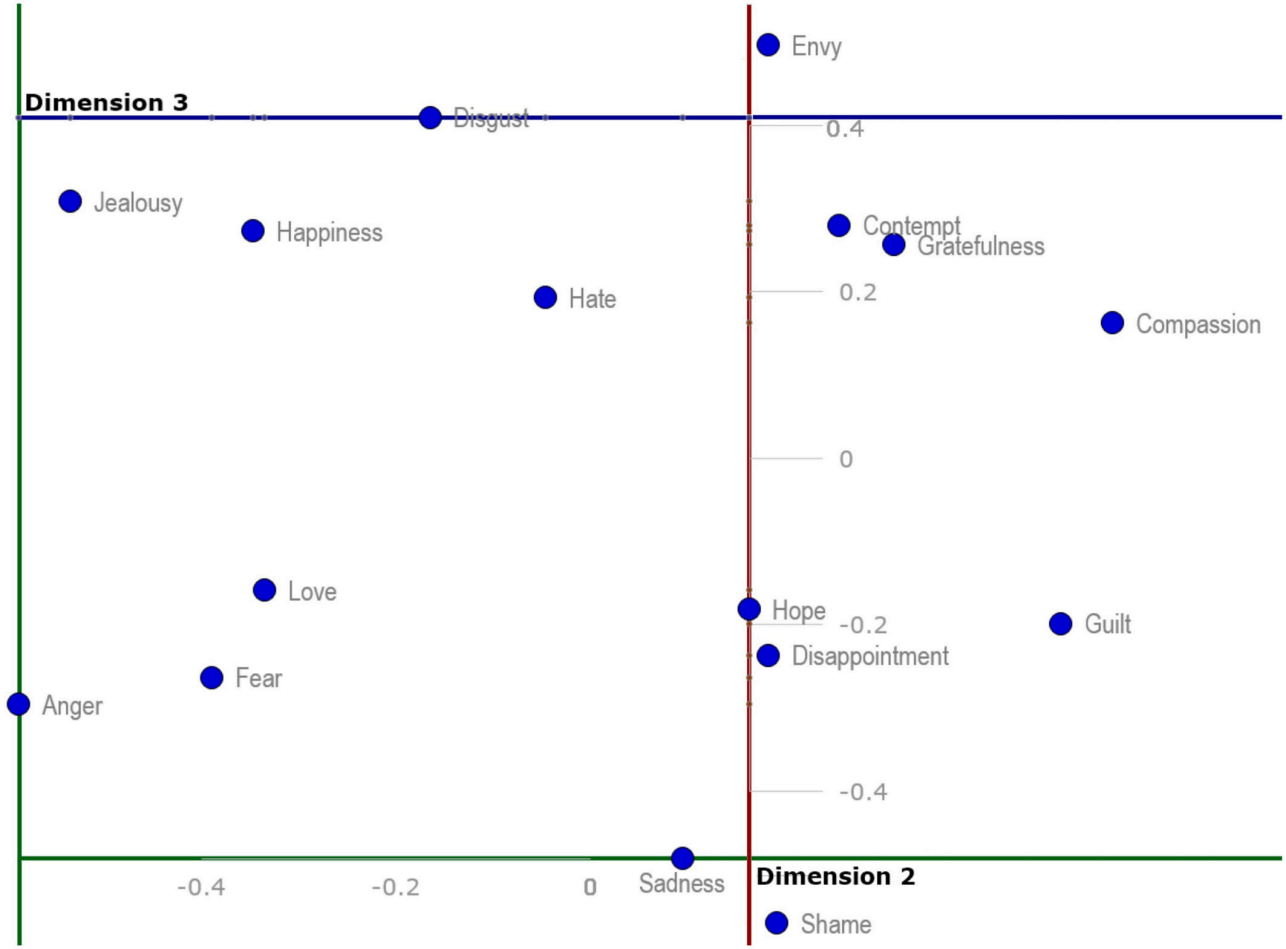
**Two-dimensional projection of discrete emotions on Dimension 2 and Dimension 3**.

A different two-dimensional representation of Dimensions 2 and 3 (Figure 3) provides a different image. The emotions are further apart and clusters are possibly less intuitive, e.g., anger, fear and love clustered together, while jealousy, happiness, disgust and possibly hate create another cluster; a third is formed by sadness and shame; a fourth by hope, disappointment and guilt; and a fifth by contempt and gratefulness. Compassion seems to again stand apart, as does envy.

**Figure 4:**
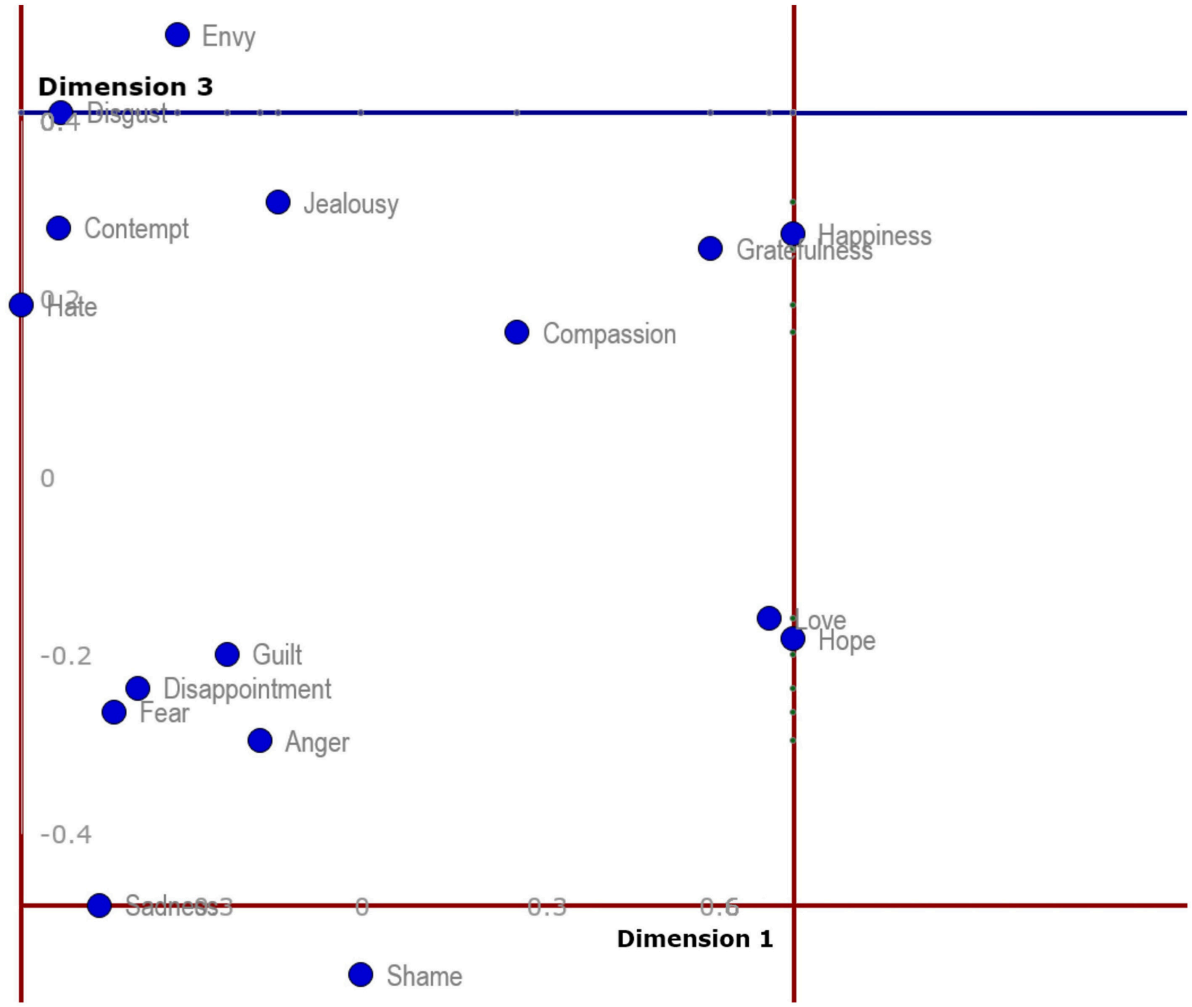
**Two-dimensional projection of discrete emotions on Dimension 1 and Dimension 3**.

The final available 2D representation provides a look at Dimensions 1 and 3 (Figure 4). In this case there is a plausible cluster containing envy, disgust, contempt, hate and jealousy on one hand; another created by guilt, disappointment, fear and anger; a third one consisting of compassion, happiness and gratefulness' and finally one of love and hope. Sadness and shame seem to be somewhat separated from the others, or possibly they create some cluster of their own.

**Figure 5:**
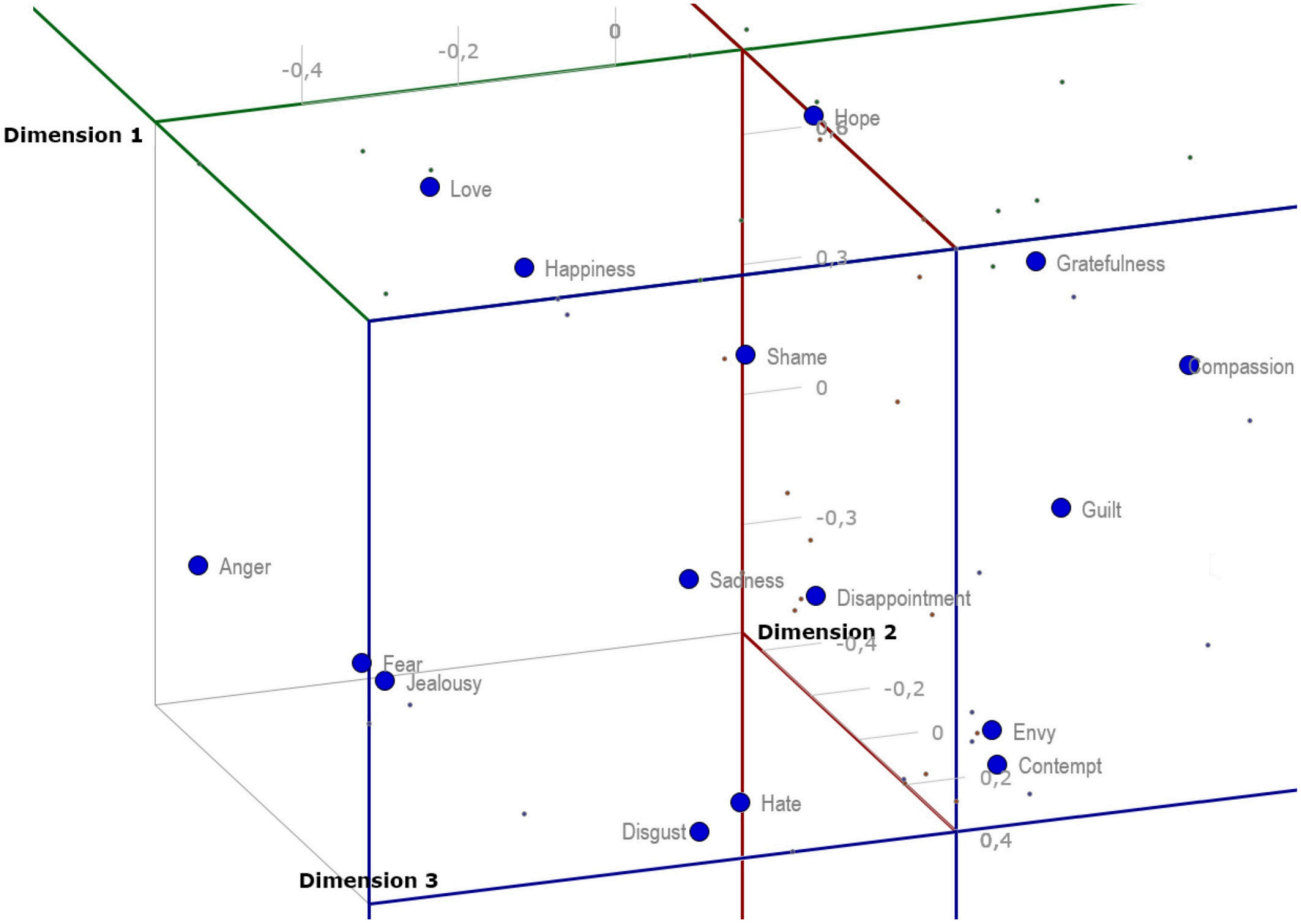
**Aggregated three-dimensional hypercube-model of emotional space based on the original four measured emotional aspects**.

A more significant separation of the emotions across all three dimensions in a 3D representation and aggregation of the original four emotional aspects can be seen in Figure 5. It seems that Dimension 1 corresponds somewhat with the original aspect of valence, where highly pleasant items (e.g., happiness, hope, love) are on the opposite side from unpleasant items (e.g., hate, disgust, sadness or fear), but it mixes with some of the original utility dimension as well. Dimension 2 seems to correspond mostly with arousal, although in reversed numbering dividing on one side highly arousing items (e.g., anger, fear, happiness, love) and on the other those which tend to be rather calm (e.g., compassion, guilt, gratefulness). Finally, Dimension 3 seems to correspond somewhat with control and partially with utility, as well. It lists on one side items that are controllable, such as envy or disgust, and on the rather uncontrollable side emotions of shame and sadness. This interpretation should not be overestimated because certain ratios of original four emotional aspects in the aggregated three-dimensional hypercube-model have not been determined.

Table 4 lists the final positions of the emotions in this model, while Table 4 lists the distances of the emotions in this MDS-constructed model.

**Table 4:** **Final positions in MDS generated mean-centered 3D model**.

**Emotion**	**Dimension 1**	**Dimension 2**	**Dimension 3**
Anger	−0.133	−0.588	−0.296
Fear	−0.386	−0.389	−0.264
Sadness	−0.412	0.095	−0.481
Happiness	0.794	−0.347	0.273
Disgust	−0.478	−0.165	0.409
Hope	0.795	0.163	−0.182
Love	0.753	−0.335	−0.159
Hate	−0.548	−0.046	0.193
Contempt	−0.483	0.256	0.280
Guilt	−0.190	0.484	−0.199
Compassion	0.315	0.537	0.162
Shame	0.043	0.192	−0.559
Gratefulness	0.652	0.312	0.256
Envy	−0.277	0.183	0.496
Disappointment	−0.345	0.183	−0.237
Jealousy	−0.101	−0.535	0.308

Similarly to the construction of the proximity matrix from the original data, we can obtain such a matrix from the MDS-results using the final positions as coordinates on x-, y-, and z- axes. The same rule applies, i.e., the lower the number in the resulting proximity matrix, the closer the two emotions are in MDS-aggregated 3D-space. The distances are listed in Table 5.

**Table 5:** **Emotional proximities in MDS-aggregated 3D space**.

**Distance (s)**	**Fear**	**Sadness**	**Happiness**	**Disgust**	**Hope**	**Love**	**Hate**	**Contempt**	**Guilt**	**Compassion**	**Shame**	**Gratefulness**	**Envy**	**Disappointment**	**Jealousy**
Anger	0.324	0.761	1.114	0.891	1.199	0.932	0.840	1.081	1.078	1.294	0.842	1.315	1.115	0.802	0.607
Fear		0.532	1.298	0.715	1.307	1.146	0.594	0.850	0.898	1.237	0.781	1.356	0.958	0.575	0.656
Sadness			1.490	0.929	1.246	1.284	0.702	0.781	0.529	1.066	0.472	1.312	0.990	0.267	1.057
Happiness				1.292	0.683	0.434	1.378	1.413	1.372	1.011	1.244	0.674	1.216	1.356	0.915
Disgust					1.441	1.366	0.256	0.440	0.935	1.087	1.156	1.236	0.411	0.746	0.538
Hope						0.501	1.411	1.363	1.037	0.699	0.842	0.484	1.269	1.142	1.237
Love							1.379	1.439	1.251	1.028	0.971	0.776	1.326	1.217	0.994
Hate								0.321	0.751	1.042	0.986	1.254	0.467	0.528	0.673
Contempt									0.607	0.854	0.993	1.137	0.308	0.541	0.880
Guilt										0.623	0.519	0.972	0.763	0.341	1.143
Compassion											0.844	0.416	0.767	0.848	1.159
Shame												1.024	1.103	0.504	1.141
Gratefulness													0.968	1.119	1.135
Envy														0.737	0.763
Disappointment															0.934

It is also possible to assess the correlation between the original 4D-space proximities matrix and the resulting MDS-aggregated 3D-space proximities matrix. The Pearson correlation is *r* = 0.758 (sig < 0.001, *N* = 120); while statistical procedures obviously reduced some amount of information, there is still very strong correlation in the resulting model.

## Discussion

The present study introduces an innovative analytical tool for approaching emotions in other than the conventional, two-dimensional paradigm. The widely accepted theoretical two-dimensional circumplex model implies that the character of most human emotions is possible to define within two general dimensions of valence and activation. The present study challenges this theoretical paradigm and provides new inspiration for further development of emotion theory.

First of all, the results of the present study indicate that various discrete emotions have more qualities perceived by individuals than only valence and activation. Participants in our study judged 16 discrete emotions in terms of valence, intensity, controllability and utility. All of these four kinds of judgments were not significantly correlated and represent independent qualities within the participants' subjective knowledge about emotions. This finding diverges from the assumption that two basic qualities, valence and activation, are sufficient for describing the prototypical character of various discrete emotions. Neither controllability nor utility were significantly correlated with valence or with intensity, and they therefore represent clearly different qualities of discrete emotions.

The independence of four kinds of dimensional measurement in our study justified the later construction of a four-dimensional model of semantic emotion space, which was then transformed into a 3D hypercube-model (Figure 5). The 3D hypercube-projection (Figure 5) was constructed based on the mutual positions of emotion prototypes that were extracted from participants' judgments of four different qualities of emotion words. This model helps to analyze and interpret the structure of semantic emotion space in a more complex manner than in cases of standard, two-dimensional analytical projections.

The present study revealed the following very important insights: (1) sources of bias when working in a two-dimensional paradigm were identified; (2) emotions that represent limits or frontiers of semantic emotion space were found; (3) no emotion prototype was settled in the central area of 3D emotion space; (4) the mutual multidimensional positions of emotional prototypes in the 3D hypercube-projection enable the multidimensional semantic similarity of used emotional prototypes to be defined more clearly than when working in a two-dimensional paradigm, (5) the mutual multidimensional positions of emotional prototypes in the 3D hypercube-model enabled the pairs of emotions that are opposite in terms of their multidimensional semantic similarity to be identified.

Most importantly, the results of the present study pointed out the risk of confusion when interpreting data based on the two-dimensional theoretical paradigm. All of the above-mentioned key insights will be discussed below.

### Sources of biases in the two-dimensional model of emotion

In the following text, our two-dimensional projections (Figures 2–4) will be used as hypothetical examples of projections from studies working with measures of qualities of discrete emotions only within two dimensions. The reader may compare the two-dimensional projections (Figures 2–4) with the 3D hypercube-projection (Figure 5) in the results section of this study. All of these projections are based on the same data set. Such comparisons revealed some potential sources of bias when working only within the two-dimensional paradigm. For example, when looking at the positions of emotion prototypes using two-dimensional projections, one may get the impression that some of the emotion prototypes are located in the central area of the space that is determined by the emotion prototypes with peripheral positions. For example, love and hate appear to be located close to the central area when using a standard two-dimensional projection of emotion prototypes on Dimension 2 and Dimension 3 (Figure 3). Similarly, compassion, guilt, disappointment, fear and anger all seem to be located close to the central area when using a two-dimensional projection on Dimension 1 and Dimension 3 (Figure 4). However, these impressions are misleading, because they are given by the limitations of two-dimensional projections, mostly by the flattening the emotion space.

### Frontiers of semantic emotion space

The positions of frontiers of semantic emotion space are exactly defined by coordinates on the x-, y-, and z- axes (Table 4). However, the question is how to interpret the positions of emotion prototypes toward the originally measured aspects, like valence, intensity, control and utility? When comparing the descriptive results (Table 1), the two-dimensional projections (Figures 3–5) and the final positions of the emotion prototypes after the MDS three-dimensional solution (Table 4), it seems that the final Dimension 1 is saturated mostly by valence and possibly partially by utility, Dimension 2 mostly by intensity of arousal, and Dimension 3 is mixed, saturated by some proportion of control and utility. However, this is only a rough estimate and not sufficiently exact. It is obvious that transforming the original four input dimensional measures (aspects) into the final three dimensions in the 3D hypercube-model (Figure 5) changed the character of the resulting three dimensions. In other words, the original judgments of participants on the dimensions of valence, intensity, control and utility saturated the resulting three dimensions (Dimension 1, Dimension 2, Dimension 3) by certain ratios.

When projecting the same data using the 3D hypercube-model, no emotion prototype is settled in central area of the three-dimensional emotion space (Figure 5). Thus, two-dimensional projections visibly flatten the space and elicit biased impressions about the positions of individual emotions. Emotions that seem to be located in the central area of the space in two-dimensional projections are indeed located far from the central area. This effect is caused by the combination of the four input dimensional judgments of participants, which set up the position in the final 3D hypercube-model. The thing is that the positions of some emotions are based on such a specific combination of judged valence, intensity, controllability and utility that their positions appear to be located close to the central area when using two-dimensional projection. Therefore, the use of two-dimensional designs in the empirical investigation of emotions is confronted with the risk of reductionist bias and oversimplification of highly complex phenomena. On the other hand, multidimensional designs and spatial, three-dimensional data projections may enrich future studies with more complex insights on the positions of semantic fields of emotional prototypes in the whole semantic emotion space.

Our results indicated that no emotions are located in the central area in the identified semantic emotion space. When interpreting this finding, we should turn our attention to the input measures, i.e., to the initial judgments of participants. Participants judged various discrete emotions according to the perceived valence, intensity, controllability and utility. Emotions generally represent states of mind that are different from emotionally-neutral or non-emotional states of mind. People usually name such emotionally-neutral states by words like “calm” or “serenity.” In the present study, the central area of the semantic emotion space did not include a prototypical semantic category for any kind of emotion. Indeed, this is not surprising. The emotional prototypes that were judged by our participants are different discrete emotions, and we would suppose that they should not yield zero or close to zero scores for their valence, intensity, controllability or utility.

### Semantic similarity of emotional prototypes

The above-mentioned source of bias was revealed when we contrasted the positions of emotion prototypes toward dimensions in the two- and three-dimensional models. Now, we turn our attention to another source of bias. It emerges when interpreting the mutual positions of discrete emotions, i.e., the semantic similarity of emotional prototypes. In the present study, anger and jealousy look to be very similar in the projection using Dimension 1 and Dimension 2 (Figure 2). However, when looking at the 3D hypercube-projection (Figure 5) or the projection using Dimension 2 and Dimension 3 (Figure 3), a very different look is available for an observer. Here it is necessary to point out that the two-dimensional projections are only another view of the same semantic emotion space.

The same bias occurs when we compare the mutual positions of, for example, sadness and contempt, or love and happiness. Some emotions look to be very similar in the two-dimensional projection, but they are indeed very different (a reader may also compare mutual proximities in the MDS-aggregated 3D space in Table 5) when a third dimension is taken into account. The two above-mentioned sources of bias indicate that research designs using traditional, two-dimensional paradigm, face a significant risk of confusion. Therefore, the combination of standard, two-dimensional projections and the 3D hypercube-projection proposed by the present study may provide a more accurate starting point for the interpretation of the results yielded in this type of research.

Further, discrete emotions that represent limits or frontiers of semantic emotion space were identified in the present study. Table 4 and Figures 2–4 enable the most extreme positions of emotion prototypes to be determined on all three dimensions of the 3D hypercube-model. These extreme positions indeed represent the extent of the three-dimensional overall semantic space constructed on the basis of participants' judgments. Emotions that border the semantic space in Dimension 1 in the positive direction are happiness, hope and love, and in the negative direction hate. The emotion that borders the semantic space in Dimension 2 in the positive direction is compassion, and in the negative direction anger. The emotion that borders the semantic space in Dimension 3 in the positive direction is envy, and in the negative direction shame. These frontiers of semantic emotion space have a very important function for personal knowledge about emotions, i.e., they constitute the personal awareness about maximal, as well as minimal, possible general qualities of emotional experience, like valence, intensity, controllability and utility. It borders the general emotionality of a person, but it does not mean that the person may not experience some emotions that would be out of this averaged semantic emotion space. First, our participants differed in their judgments of emotional qualities (see SDs in Table 1). Second, the judgments of participants were based on the most frequent experiences with particular emotions in their personal histories. Some extreme and non-frequent emotional episodes may sometimes occur, but it is unlikely that such experiences would influence the judgment of an emotion prototype radically, because personal knowledge about emotions is shaped in the long-term course of emotional development and maturation. On the other hand, merely different levels of emotional maturation as well as differences in emotional personal traits may be some of the factors influencing the participants' judgments, and therefore, also the interpersonal differences in the extent of semantic emotion spaces (see SDs in Table 1).

### Limitations

The innovative analytical tool introduced here has some limitations. One of them is the problematic graphical depiction of four-dimensional emotion space. The standard Euclidian geometric space does not enable us to project all four originally measured aspects proportionally. The graphical depiction is a result of the MDS three-dimensional solution used and the inspiration for future methodological shifts is a hypothetical projecting tool that would be more suitable for working with more than three-input dimensional measurements (e.g., an animated tesseract). Given the complexity of the human emotional experience, it is the way to move the field forward and not to permit some superfluous reduction of the phenomena.

Another limitation of the present study is that emotionally-neutral semantic categories were not included in the research design. Participants did not judge emotion-neutral semantic categories such as calm or serenity. This limitation meant that the core of the semantic emotion space could not be clearly identified from the data. Also, the general orientation of semantic emotion space toward other non-emotional semantic fields is therefore hardly accessible. Future research in this field should consider such emotionally-neutral semantic categories to avoid the above-mentioned shortcomings.

## Conclusion

The results of the present study pointed out to some limitations of the two-dimensional paradigm in the psychological theory of emotions. Contrasting two-dimensional and three-dimensional projections helped us to identify some sources of bias that may increase the risk of reductionism when approaching such complex phenomena as human emotions. The discussion of the results indicated that the 3D hypercube-projection may provide researchers with more in-depth insights into the overall structure of semantic emotion space than the traditional two-dimensional projections widely used in this field.

As mentioned throughout the Discussion section, the findings presented here signify very relevant implications for the contemporary theory of emotions. Of course, we do not want to reject the widely used circumplex model (Russell and Lemay, 2000). This model has been verified by many other empirical studies in the past, and we believe that our study contributes a new piece of knowledge to current theoretical discourse. The results of the present study provide a slightly different look at the phenomena and may inspire future researchers to continue in the discussion about the dimensionality of human emotional experience. Some alternative approaches, such as the theory of multi-dimensional emotional experience (Trnka, 2013), may be taken into account.

The current application of Sokolov and Boucsein's (2000) theoretical concept of the hypersphere in emotion research also opens up new and exciting questions for future studies. For example, what is the shape of such a hyperspace? May we even think about visualization of intangible data in emotion research (Murín, 2014)? Is there some kind of symmetry in multidimensional semantic emotion space? Or, is it possible to speak about a shape when exploring a phenomenon that is probably even more multi-dimensional than we are currently able to depict? These are rather philosophical questions and answering them is far beyond the scope of the present study. But, according to a famous bon mot of quantum physicist Niels Bohr we conclude that “The question may not be, whether a theory is too crazy, but whether it is crazy enough”!

## Author contributions

All authors revised the manuscript critically, approved the final version of the manuscript and agreed with all aspects of the work. RT conceptualized the research design and drafted Introduction and Discussion sections. AL conducted the data analysis and drafted Methods and Results sections. MK gathered the data. KB and PT worked on the conception of the work, the experimental design, and the interpretation of data.

### Conflict of interest statement

The authors declare that the research was conducted in the absence of any commercial or financial relationships that could be construed as a potential conflict of interest.
